# Global longitudinal strain is a hallmark of cardiac damage in mitral regurgitation: the Italian arm of the European Registry of mitral regurgitation (EuMiClip)

**DOI:** 10.1186/s12947-019-0178-7

**Published:** 2019-11-21

**Authors:** Ciro Santoro, Maurizio Galderisi, Roberta Esposito, Agostino Buonauro, Juan Manuel Monteagudo, Regina Sorrentino, Maria Lembo, Covadonga Fernandez-Golfin, Bruno Trimarco, Josè Luis Zamorano

**Affiliations:** 1 Department of Advanced Biomedical Science, Interdepartmental Laboratory of Cardiac Imaging, Federico II University Hospital, V. S. Pansini 5, bld 1, 80131 Naples, Italy; 2 Department of Cardiology, University Hospital Ramon y Cajal, Madrid, Spain

**Keywords:** Mitral regurgitation, Ejection fraction, Global longitudinal strain, Left atrial volume, Pulmonary hypertension

## Abstract

**Background:**

The search for reliable cardiac functional parameters is crucial in patients with mitral regurgitation (MR). In the Italian arm of the European Registry of MR, we compared the ability of global longitudinal strain (GLS) and left ventricular (LV) ejection fraction (LVEF) to detect cardiac damage in MR.

**Methods:**

Five hundred four consecutive patients with MR underwent a complete echo-Doppler exam. A total of 431, 53 and 20 patients had degenerative, secondary and mixed MR, respectively. The main echocardiographic parameters, including LV and left atrial (LA) size measurements, pulmonary artery systolic pressure (PASP) and GLS were compared between patients with mild MR (*n* = 392) vs. moderate to severe MR (*n* = 112).

**Results:**

LVEF and GLS were related one another in the pooled population, and separately in patients with mild and moderate/severe MR (all *p* < 0.0001). However, a certain number of patients were above the upper or below the lower limits of the 95% confidence interval (CI) of the normal relation in the pooled population and in patients with mild MR. Only 2 patients were below the 95% CI in moderate to severe MR. After adjusting for confounders by separate multivariate models, LVEF and GLS were independently associated with LV and left atrial size in the pooled population and in mild and moderate/severe MR. GLS, but not LVEF, was also independently associated with PASP in patients with mild and moderate to severe MR.

**Conclusions:**

Both LVEF and GLS are independently associated with LV and LA size, but only GLS is related to pulmonary arterial pressure. GLS is a powerful hallmark of cardiac damage in MR.

## Introduction

The management of mitral regurgitation (MR), the most common valvular heart disease in the developed world [1], is mainly based on symptoms and signs of left ventricular (LV) dysfunction [2, 3]. Nevetherless, physicians face difficulties in detecting subtle progression of symptoms related to MR deterioration. Patients might unwittingly limit their physical activity because of worsening exercise capacity and overt symptoms. In this clinical setting, physicians are requested to perform a strict follow-up mainly using echocardiographic parameters to detect early signs of LV failure. Nowadays, standard echocardiographic parameters, mainly two-dimensional derived LV ejection fraction (LVEF), are the most commonly used for evaluating LV function and prognosis in patients with MR [4]. However, despite a preserved LVEF, patients with chronic MR often hide an underlying myocardial contractility impairment [5]. LVEF does not take into account the amount of blood flow pushed back in the low-pressure left atrial (LA) cavity, which does not contribute to the effective stroke volume. Accordingly, patients with chronic MR and impaired LVEF may already have developed an irreversible, undiagnosed myocardial damage. The efforts of cardiologists should be addressed to search for alternative parameters that could unmask subtle, insidious changes in LV contraction.

LV deformation as assessed by speckle tracking echocardiography (STE) has proven to detect subclinical cardiac involvement and have prognostic value in different pathologic conditions [6, 7]. Analysis of myocardial LV deformation by STE also identified early LV dysfunction in patients with asymptomatic severe MR. GLS have demonstrated to predict 5-years all-cause mortality risk in patients with acute heart failure independently from LVEF values [8]. LV global longitudinal strain (GLS) at rest is also a strong predictor of long-term prognosis in a large population of asymptomatic patients with severe primary MR and preserved LVEF [9].

The purpose of the present study was to analyse differences between GLS and LVEF in detecting myocardial damage in patients with different etiology and mechanism of MR, enrolled in the Italian arm of the European Registry of MR (EuMiClip) [10]. Secondarily, we aimed to explore potential relationships between longitudinal deformation alteration and echocardiographic structural and functional parameters in patients with MR of different degree.

## Methods

### Study population

Over a 3-month period (March–June 2016), all consecutive patients with MR were prospectively recruited from the Italian arm of the European Registry of MR (EuMiClip). The registry protocol as well as the enrolment criteria have already been described [10]. Briefly, we included patients with different degree of MR and different etiology, among those referred to our echo-lab (Interdepartmental Laboratory of Cardiac Imaging, Federico II University Hospital, Naples, Italy). All patients gave their informed consent. Physical examination was performed and clinical history was collected. Complete baseline transthoracic echocardiographic studies, including STE acquired in three apical views, were computed. Blood pressure (BP) was measured at the end of the echocardiographic examination. Patients with trivial MR and those with suboptimal image quality that precluded standard echocardiographic examination and/or longitudinal strain determination were excluded.

### Echocardiographic exam

All echocardiographic examinations were performed according to the general procedures of EuMiClip Registry [10] and EACVI standardization of the echo report [11] on a Vivid E9 ultrasound machine (GE Healthcare, Horten, Norway), using a 2.5 MHz transducer with harmonic capability. All the echo parameters were obtained as the average of three consecutive cardiac cycles in patients with sinus rhythm and of five consecutive cardiac cycles in presence of atrial fibrillation. LV dimensions were calculated according to the 2015 ASE/EACVI recommendations for chamber quantification [12], and LVEF was derived by measuring LV end-diastolic and end-systolic volumes with the biplane method. LA volume was indexed for body surface area (LAVi) [12]. Pulmonary artery systolic pressure (PASP) was derived from the tricuspid regurgitation velocity gradient and inferior vena cava size and reactivity as recommended by the 2015 ESC/ERS guidelines [13]. In addition to the general procedures of the EuMiClip Registry, in the Italian arm of the registry we assessed GLS using STE according to procedures of our laboratory [14]. In particular, peak negative longitudinal strain was measured from 6 segments in each of the three apical views (long-axis, 4- and 2-chamber), and GLS calculated as the average of individual peak strain before aortic valve closure. GLS values were considered positive (sign +) to strengthen the clinical meaning: the higher the values, the better is the strain deformation. Reproducibility of GLS in our laboratory has been recently reported [15].

MR was firstly assessed by color Doppler derived regurgitant jet area as recommended [16]. More than mild MR was computed using quantitative methods, including vena contracta width and/or proximal isovelocity surface area (PISA) when feasible. MR was classified by etiology as “primary” if due to intrinsic valve disease, “secondary” if no valve structural abnormalities were evident, or “mixed” if both LV remodeling with mitral valve tethering and structural valve lesions were present. Causes of primary MR (e.g. degenerative, rheumatic, Barlow’s disease, endocarditis) and/or secondary MR were listed (e.g. ischemic, dilated cardiomyopathy). Information on jet direction (central, medial, lateral, and complex) and presence and degree of calcification and valve segment involved (segment 1, 2 or 3) were collected in patients with moderate to severe MR. In these patients, mechanisms of MR based on the Carpentier’s classification of leaflet motion were also reported: type I, normal leaflet motion; type II, excessive leaflet motion; type III, a-restricted leaflet opening, b-restricted leaflet closure [17]. Concomitant hemodynamically significant valve heart diseases were also searched and reported.

### Statistical analysis

Statistical analyses were performed using SPSS package, release 12.0 (SPSS Inc., Chicago, Illinois, USA). Normal distribution of data was checked using the Kolmogorov–Smirnov test. Differences between variables were assessed using the paired t test or one-factor ANOVA as appropriate. Chi-square analysis was used to calculate inter-group rate difference of given parameters. Univariate correlates of a given variable were evaluated by least squares linear regression with computation of means and 95% confidence intervals (CI). Multiple linear regression analysis was performed to identify independent correlates of a given variable, after adjusting for confounders. Multicollinearity was assessed by computation of in-model variance inflation factor. Intra- and inter-observer variability of LA volume, LV end-diastolic volume, end-systolic volume and PASP was assessed by calculating intra-class correlation coefficient (ICC) within 95% confidence intervals (CI). The null hypothesis was rejected at 2-tailed *p* < 0.05.

## Results

### Distribution and echocardiographic features

Among 1820 patients referring to our echo lab for different cardiac pathologies during the 3 months recruitment period, 504 (27.7%) had evidence of MR of variable degree. Patients’ characteristics are summarized in Table 1. The mean age was 59.6 years, with a larger prevalence of women; 21 patients (4%) showed atrial fibrillation at the time of the echocardiographic exam. 109 patients were obese (body mass index ≥30 kg/m^2^) and 161 hypertensive (BP ≥140/90 mmHg) (data not shown in table).

**Table 1:** Study population characteristics

Variable	Mean ± SD	Range
Sex (M/F)	275 / 229	–
Age (years)	59.6 ± 14.3	15–93
Body Weight (Kg)	72.9 ± 15.2	26–164
Height (m)	1.65 ± 0.10	1.10–2.38
BMI (Kg/m^2^)	26.6 ± 4.9	12.1–50.6
Heart Rate (bpm)	69.5 ± 12.3	45–146
Systolic BP (mmHg)	129.0 ± 17.0	85–200
Diastolic BP (mmHg)	77.1 ± 9.3	50–110
Atrial Fibrillation (n/%)	21/4%	–

*BP* Blood pressure, *BMI* Body mass index

Classification and degree of MR, along with the main anatomical features, are reported in Table 2. The majority of MR patients (85.5%) had a primary etiology (59 with moderate to severe MR. The prevalence of secondary MR was 10.5% (*n* = 53), with a rate of moderate to severe MR of 66%, while very few patients (4%, *n* = 20) exhibited MR with mixed etiology (18 with moderate to severe MR). Prevalence of concomitant hemodynamically significative (more than mild) heart valve diseases are reported. Tricuspid regurgitation was the most frequent (11% of the overall population), followed by aortic regurgitation.

**Table 2:** Classification of MR based on etiology, mechanism, degree and frequencies of concomitant valve disease in the study population

Variable		Frequencies	Percentage (%)
Etiology	Primitive	431	85.5
	Secondary	53	10.5
	Mixed	20	4
Degree	Mild	392	77.8
	Moderate	104	20.6
	Severe	8	1.6
Mechanism ^a^(Carpentier’s classification)	Type I	14	12.5
	Type II	4	2.7
	Type III a	53	46.4
	Type III b	41	34.8
Concomitantsignificative valve disease	Mitral Stenosis	1	0.2
	Aortic Regurgitation	22	4
	Aortic Stenosis	7	1
	Tricuspid regurgitation	55	11

^a^Only for patients with moderate to severe MR

LVEF was 59.9 ± 7.4% (range 28–77%), LAVi 31.6 ± 12.5 ml/m^2^ (range 12.8–101.3), estimated PASP 19.8 ± 3.7 mmHg (range 5–28 mmHg), and GLS 19.8 ± 3.7% (5–28%). The feasibility of GLS was almost optimal, it being quantifiable in 460 of the 504 enrolled patients (91%).

**Fig. 1:**
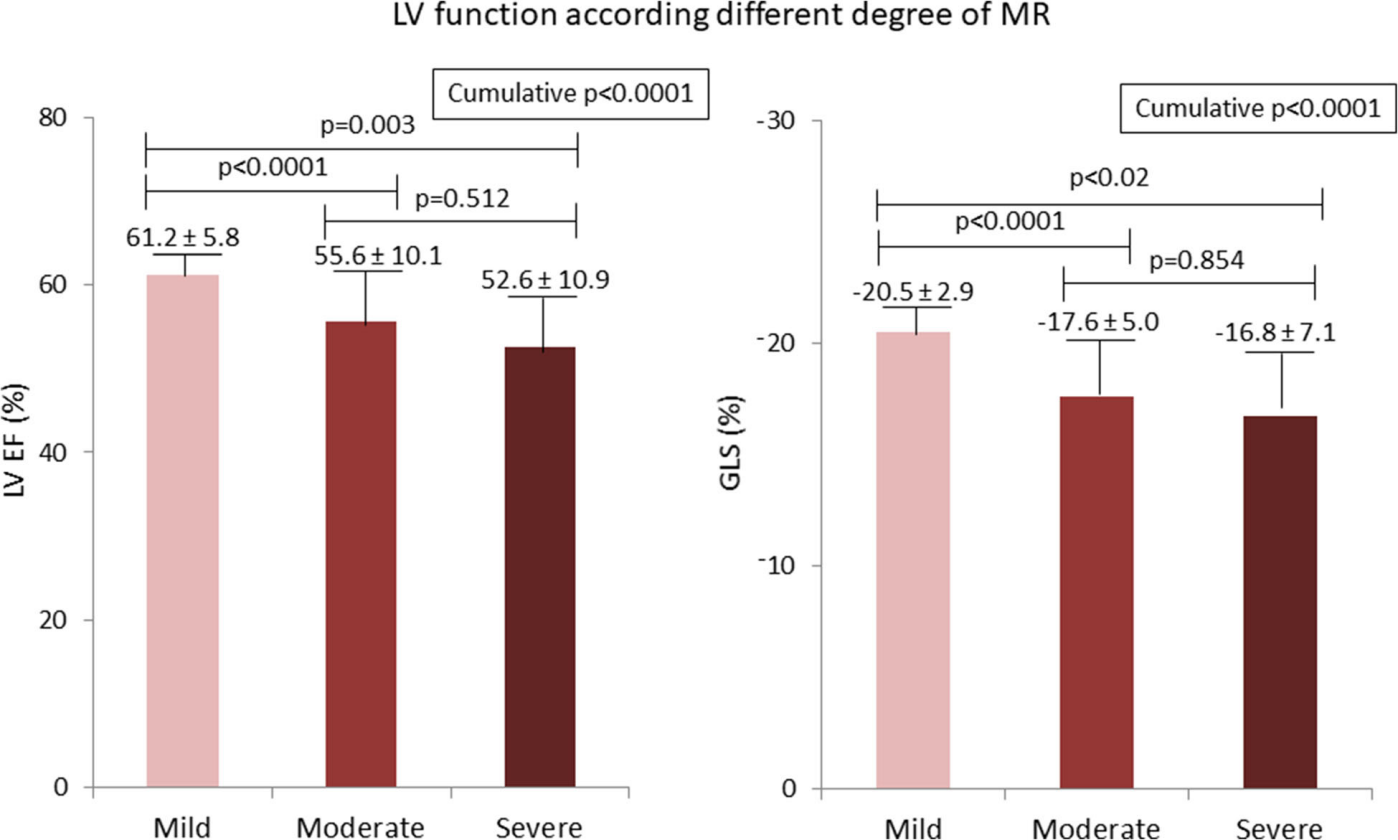
Differences in left ventricular ejection fraction (LVEF) and global longitudinal strain (GLS) by MR degree

Figure 1 depicts the differences in LVEF and GLS according to MR degree. Both LVEF and GLS were lower in moderate than in mild MR, whereas there was no significant difference between moderate and severe MR. Greater LV size and LA volume, lower LVEF and GLS, and higher PAPS (all *p* < 0.0001) were also observed in moderate to severe MR compared to mild MR (Table 3).

**Table 3:** Differences of echocardiographic parameters between patients with mild vs. moderate to severe MR

Variable	Mild MR *n* = 392	Moderate to severe MR *n* = 112	*p*
LV end-diastolic diameter (mm)	49.3 ± 6.0	53.4 ± 7.8	<0.0001
LV end-systolic diameter (mm)	31.6 ± 6.0	36.2 ± 9.6	<0.0001
LV end-diastolic volume (ml)	86.9 ± 27.9	104.2 ± 38.6	<0.0001
LV end-systolic volume (ml)	33.9 ± 13.5	49.9 ± 28.3	<0.0001
LV EF (%)	61.2 ± 5.8	55.4 ± 10.1	<0.0001
GLS (%)	20.5 ± 3.0	17.5 ± 5.2	<0.0001
LAVi (ml/m^2^)	28.5 ± 8.5	42.6 ± 17.3	<0.0001
Estimated PASP (mmHg)	29.6 ± 7.5	36.9 ± 11.1	<0.0001

*GLS* Global longitudinal strain, *LAVi* LA volume index, *LV* Left ventricular, *PASP* Pulmonary arterial systolic pressure

### Univariate correlations and independent associations

**Fig. 2:**
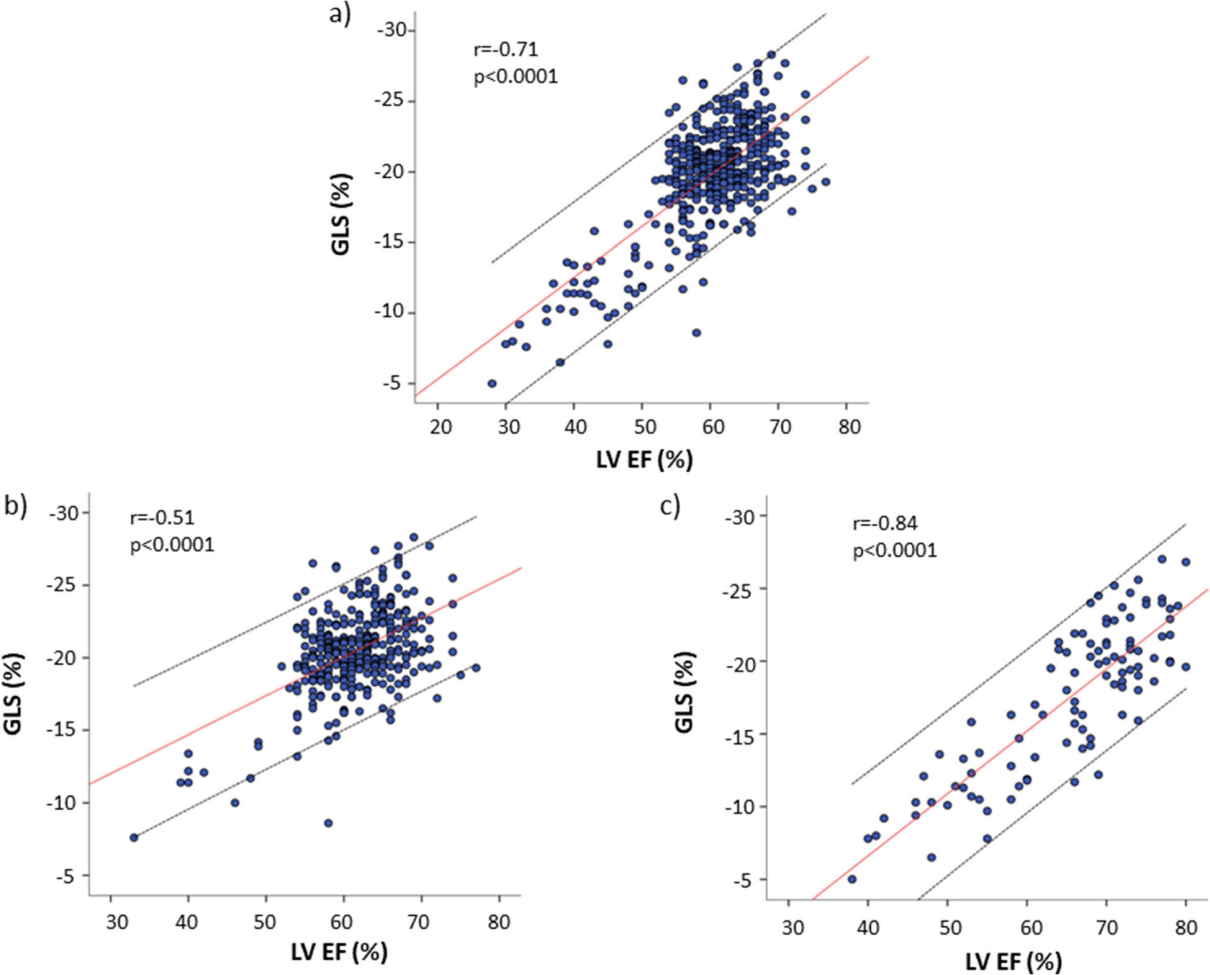
Scatterplot showing the univariate correlation of indexes of LV systolic function and pulmonary artery systolic pressure. Correlation between (**a**) LVEF, (**b**) GLS with PASP in the overall population. Subgroup analysis showing correlation between (**c**) LVEF, (**d**) and GLS with PASP in patients with moderate to severe MR

LVEF and GLS were positively related one another in the pooled population (r = 0.71), and in both sub-analysis in patients with mild (r = 0.51) and moderate to severe MR (r = 0.84) (all p < 0.0001) (Fig. 2). However, when considering the 95% CI of the normal relation, particularly in the subgroup with mild MR, a certain number of MR patients were above the upper or below the lower limits. Differently, only 2 patients were below the 95% CI of the normal relation in the subgroup with moderate to severe MR.

**Fig. 3:**
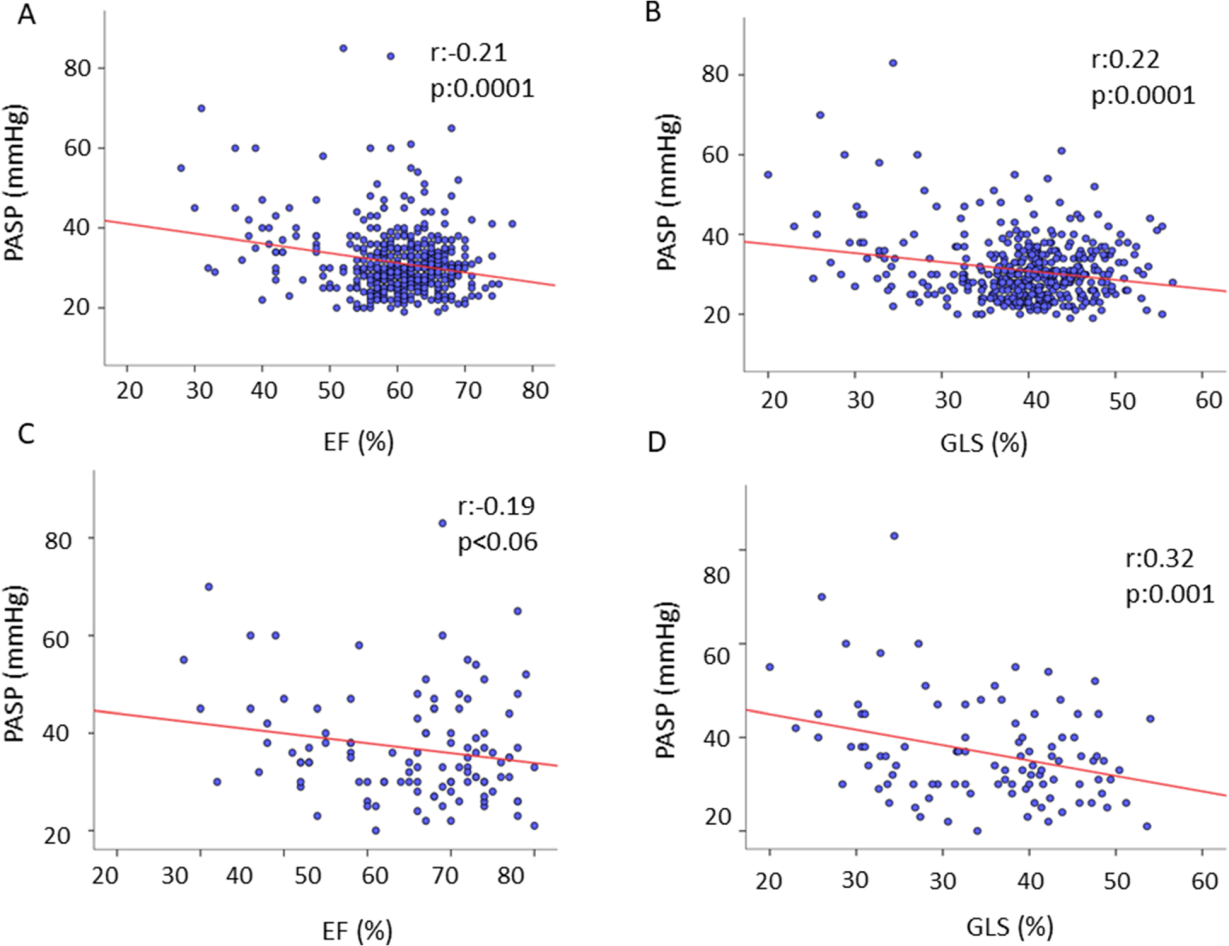
Relation between left ventricular ejection fraction (LVEF) and global longitudinal strain (GLS) in the pooled population (**a**), and in patients with mild (**b**) and moderate to severe mitral regurgitation (MR) (**c**). Positive relations are seen in all the three groups, but data points for a certain number of MR patients are above the upper and below the lower limit of the 95% confidence interval (CI) of the normal relation in (**a**) and (**b**) (parallel dotted lines). Conversely, the positive relation in moderate to severe MR (**c**) exhibits only 2 patients below the 95% CI

Figure 3 depicts univariate correlations of both LVEF and GLS in the pooled MR population (top) and in patients with moderate to severe MR (bottom). Of note, both LVEF and GLS correlated negatively with PASP in the entire study population but, in presence of moderate to severe MR only the correlation with GLS remained significant.

Table 4 shows independent associations of LVEF and GLS, after adjusting for age, body mass index, heart rate and systolic BP obtained by multiple regression analyses in the pooled population and, separately, in patients with mild and moderate to severe MR. In the pooled population, both parameters were independently and negatively associated with LV diameters and volumes as well as with LA size and PASP. In the subgroup with mild MR, only GLS maintained an independent association with PASP. In patients with moderate to severe MR, GLS had a more significant association with LAVi than LVEF (*p* < 0.005 and *p* = 0.008, respectively) and showed a significant association with PASP (*p* = 0.004), which was not evident for LVEF (*p* = 0.09).

**Table 4:** Multivariate associations of LV EF and GLS

Dependent Variable	Covariate	β coefficient	*p* value
a. in the pooled patients MR			
LV EF	LV end-diastolic diameter	− 0.41	<0.0001
	LV end-systolic diameter	−0.54	<0.0001
	LV end-diastolic volume	−0.40	<0.0001
	LV end-systolic volume	−0.71	<0.0001
	LA diameter	−0.25	<0.0001
	LAVi	−0.38	<0.0001
	PASP	−0.18	<0.0001
GLS ^a^	LV end-diastolic diameter	−0.40	<0.0001
	LV end-systolic diameter	−0.54	<0.0001
	LV end-diastolic volume	−0.40	<0.0001
	LV end-systolic volume	−0.60	<0.0001
	LA diameter	−0.32	<0.0001
	LAVi	−0.41	<0.0001
	PASP	−0.19	<0.0001
b. In patients with mild MR			
LV EF	LV end-diastolic diameter	− 0.24	<0.0001
	LV end-systolic diameter	−0.37	<0.0001
	LV end-diastolic volume	−0.22	<0.0001
	LV end-systolic volume	−0.62	<0.0001
	LA diameter	−0.05	0.417
	LAVi	−0.22	<0.0001
	PASP	−0.02	0.735
GLS ^a^	LV end-diastolic diameter	−0.20	<0.0001
	LV end-systolic diameter	−0.35	<0.0001
	LV end-diastolic volume	−0.25	<0.0001
	LV end-systolic volume	−0.45	<0.0001
	LA diameter	−0.13	0.04
	LAVi	−0.23	<0.0001
	PASP	−0.18	=0.003
c. In patients with moderate to severe MR			
LV EF	LV end-diastolic diameter	−0.50	<0.0001
	LV end-systolic diameter	−0.59	<0.0001
	LV end-diastolic volume	−0.46	<0.0001
	LV end-systolic volume	−0.72	<0.0001
	LA diameter	−0.21	0.03
	LAVi	−0.27	0.008
	PASP	−0.17	0.09
GLS ^a^	LV end-diastolic diameter	−0.47	<0.0001
	LV end-systolic diameter	−0.59	<0.0001
	LV end-diastolic volume	−0.41	<0.0001
	LV end-systolic volume	−0.61	<0.0001
	LA diameter	−0.25	<0.02
	LAVi	−0.29	<0.005
	PASP	−0.30	=0.004

Abbreviations as in Table 4

^a^Values of GLS considered as “positive” (sign +) to build the associations in order to strengthen their clinical meaning: the higher values the better strain deformation independent on the plus/minus sign

Reproducibility analyses of standard echocardiographic parameters were performed in 20 of our study patients (Additional file 1: Table S1): notably, both intra- and inter-observer reproducibility of GLS was substantially higher than that of LVEF.

## Discussion

In the present study, during a 3-month assessment period of the European Registry of MR in our general hospital echocardiographic laboratory, 27.7% of patients were diagnosed MR of variable degree. GLS measurement was able to provide valuable clinical information for the detection of target organ damage in this clinical setting. Notably, GLS was obtained in 91% of MR patients, thus demonstrating an almost optimal feasibility. The head-to-head comparison between LVEF and GLS showed that (i) LVEF and GLS are both lower in moderate and severe than in mild MR, (ii) the relation between LVEF and GLS appears to be flatter in mild than in moderate to severe MR, and (iii) GLS, but not LVEF, is independently associated with increased PASP in mild and in moderate to severe MR.

LVEF is a reference parameter for LV systolic function in the clinical practice and is widely used also in patients with MR to guide decision-making, including the choice for surgery [16], and to predict postoperative LV dysfunction [18–20]. However, due to its strong preload dependence, LVEF is poorly sensitive in detecting early abnormalities of LV contractility [21]. In contrast, despite also being preload-dependent [22], GLS was shown to have significantly better ability to detect early impairment of LV systolic function in both primary [23] and secondary MR [24] and to predict functional capacity in asymptomatic patients with MR and preserved LVEF [25]. GLS is also predictive of post-operative LV dysfunction in MR patients undergoing mitral valve surgery [26, 27] or mitral valve repair [28, 29]. Moreover, LV longitudinal function is the main determinant of mortality in patients with primary MR, whereas resting/exercise LVEF and MR degree at rest have no prognostic value [30].

In the present study, as expected, LVEF and GLS were both lower in moderate to severe MR compared with mild MR, showing a highly significant positive relationship in the pooled population. Indeed, since LVEF and GLS represent a relative change in volume and length respectively, from a pure mathematical viewpoint it is largely expectable that these two parameters do not correlate in linear fashion [31]. Consistently, in our mild MR subgroup, which mainly includes primary etiology MR and therefore also a lower degree of LV dysfunction, the linear relation between LVEF and GLS was flatter, with few patients with overly higher GLS and a substantially greater number of individuals with disproportionately reduced GLS, in comparison with LVEF. Accordingly, in this subpopulation the proportion of patients with low GLS was greater than that of patients with reduced LVEF. Although we cannot know if GLS reduction in this our subpopulation occurs because MR itself or the concomitance of other cardiovascular risk factors, this finding strongly, albeit indirectly, supports the ability of GLS to detect early, subclinical abnormalities of LV systolic function in mild MR, not identifiable by LVEF itself.

The incremental diagnostic value of GLS was lost in patients with moderate to severe MR, in whom the slope of the regression line of the relation between these two parameters was as much stronger and no significant differences was found in the proportion of patients with reduced GLS and reduced LVEF. It is conceivable that, with the increase of MR severity, the greater burden of loading changes could tend to equalize the relation between GLS and LVEF which becomes more linear.

Additional insights were provided by the univariate and multivariate associations of both LVEF and GLS with the other echocardiographic measurements used in the EuMiClip Registry. In separate multiple linear regression analyses, body mass index as an expression of LV preload, systolic BP accounting for afterload, heart rate and age were chosen as potential confounders. By adjusting for these variables, both LVEF and GLS were independently and strongly associated with indices of preload (LV end-diastolic diameter and volume, LAVi) and afterload (LV end-systolic diameter and volume) in the pooled population and, separately, in mild and moderate to severe MR, well reflecting cardiac pathophysiology of MR. The association of both LVEF and GLS with PASP was strongly significant in the pooled model, whereas interesting discrepancies were observed when groups were analyzed separately: the association of GLS with PASP remained statistically significant, whereas that of LVEF with PASP disappeared in both the subgroups with mild and moderate to severe MR. During MR progression, passive backward transmission of elevated LA pressure leads to post-capillary pulmonary artery pressure elevation. In the 2016 ASE/EACVI recommendations for the evaluation of LV diastolic function [32], non-invasive estimation of PASP by tricuspid regurgitation velocity is considered one of the key parameters for identifying patients with increased LV filling pressures (LVFP). Additionally, PASP estimation provides incremental prognostic usefulness to standard clinical predictors in patients with primary MR [33], including those undergoing surgery for degenerative mitral valve disease [34, 35]. Noteworthy, LVFP increase and subsequent PASP elevation in MR are mediated by LA enlargement, which is a function of elevated preload and LA pressure as well, and of myocardial fibrosis occurring in the late disease stages [36, 37]. Accordingly, LA size of our patients was greater in moderate to severe than in mild MR, and the magnitude of the independent association with LAVi was slightly greater for GLS than for LVEF.

### Limitations

As a subanalysis of the EuMiClip Registry [10], patients with a broad spectrum of indications for echocardiographic exam were included, thus different comorbidity could act as confounding factors altering the pathological path of MR disease. Comparison analysis on GLS data with the other arms of the EuMiclip registry was not achievable due to intervendor variability. This limited the study analysis to the sole population included in our laboratory and hindered separate subanalysis according to mechanism and etiology of MR, due to low statistical power. Indeed, ‘primary’ and ‘secondary’ MR are distinctly different diseases in their etiologies, pathophysiology and in therapies and it is not easy to make common considerations between these two clinical conditions.

Furthermore, the small sample size of patients with severe MR is consistent with the characteristics of patients referred to our general hospital echocardiographic laboratory, where only few pre-surgical MR patients are examined. Laboratories connected with cardiac surgery departments can easily collect data from larger numbers of patients with severe MR that are candidate to invasive diagnostic and interventional procedures.

Finally, although the last chamber quantification recommendations propose possible reference normal values of GLS [12], the definition of “normal” GLS in subjects without cardiovascular disease remains to be elucidated. Some studies have described the higher-than-normal values of GLS in chronic severe MR, as the pathophysiological condition (reduced afterload and increased preload) results in a state of hypernormal LV function [38]. These findings also suggests that GLS is a load-dependent and should be therefore corrected for LV volumes. Accordingly, in the present study we could not indicate a clear cut-off point of GLS to be considered as definitively normal in the setting of MR.

## Conclusions

**Fig. 4:**
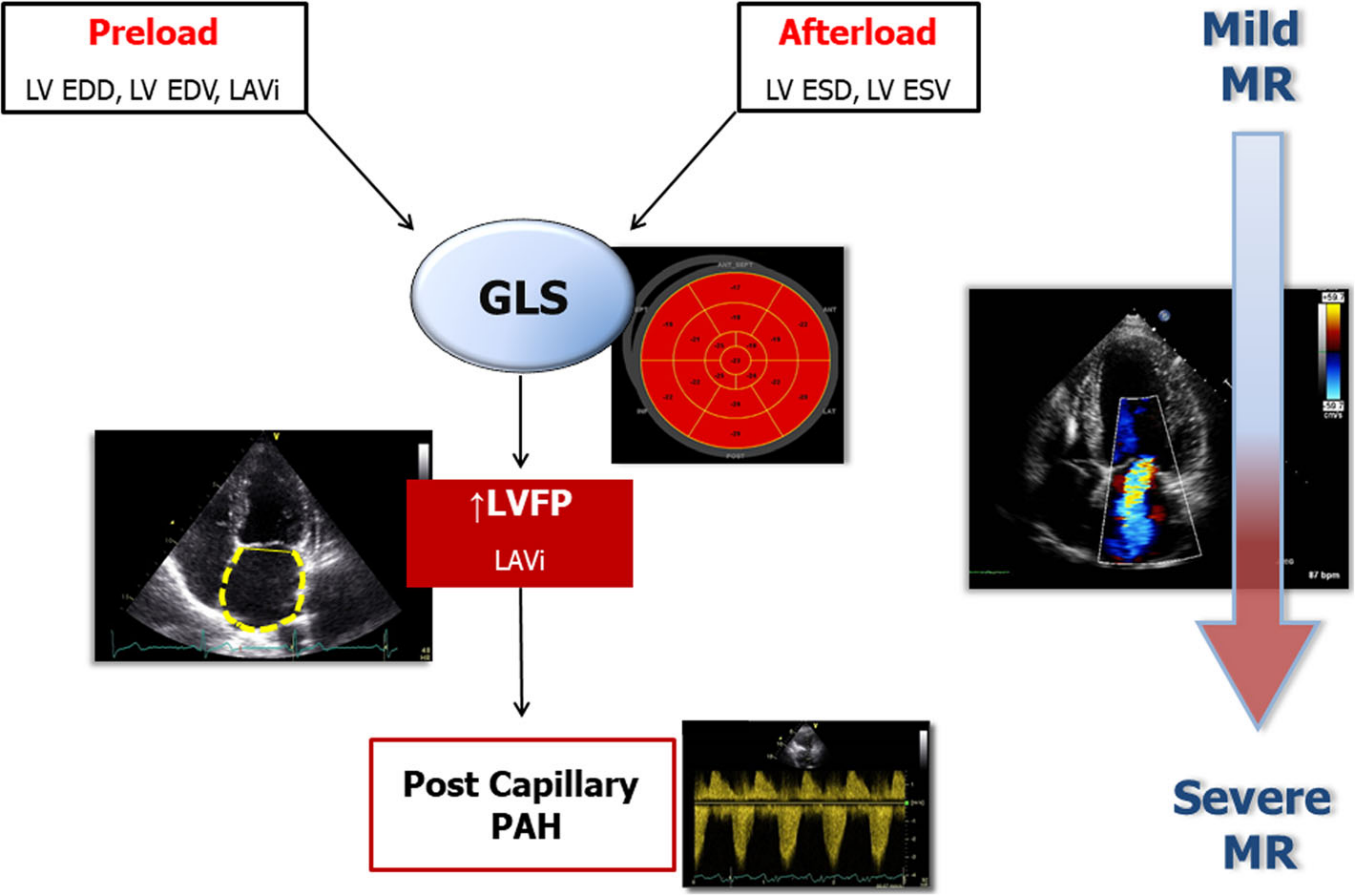
Schema depicting the central role of global longitudinal strain (GLS) in detecting cardiac damage in mitral regurgitation (MR). LAVi = left atrial volume index; LVEDD = left ventricular end-diastolic diameter; LVEDV = left ventricular end-diastolic volume; LVESD = left ventricular end-systolic diameter; LVESV = left ventricular end-systolic volume; LVFP = left ventricular filling pressure; PAH = pulmonary arterial hypertension

In the Italian arm of the EuMiClip Registry, LVEF and GLS are related one another but their relation appears to be flatter in the group with mild MR. In this subgroup, GLS shows a greater ability in detecting impairment of LV systolic function. LVEF and GLS are both independently associated with the main echo parameters of LV and LA size, but only GLS is related to PASP in mild and moderate to severe MR. The recognized optimal feasibility and reproducibility of GLS, substantially greater than that of LVEF [39, 40], is also confirmed in the present study. Our findings highlight therefore the central role of GLS as a hallmark of cardiac damage in patients with MR (Fig. 4). GLS is more sensitive than LVEF in detecting early impairment of LV systolic function in mild MR. Differently from LVEF, GLS is also a clue of elevated LA pressure and post-capillary pulmonary hypertension as an expression of increased LV filling pressures, both in mild and moderate to severe MR. GLS can be useful across all the spectrum of MR patients for guiding management, stratifying prognosis, and possibly establishing the appropriate timing for interventional procedures.

## Supplementary information

**Additional file 1: Table S1.** Reproducibility of standard echocardiographic parameters

## Data Availability

Availability of data and material under request.

## Abbreviations

ASE: American Society of Echocardiography
CI: Confidence interval
EACVI: European Association of Cardiovascular Imaging
GLS: Global longitudinal strain
ICC: Intraclass correlation coefficient
LA: Left atrial
LAVi: Left atrial volume index
LV: Left ventricular
LVEF: Left ventricular ejection fraction
MR: Mitral regurgitation
PASP: Pulmonary arterial systolic pressure
PISA: Proximal isovelocity surface area
STE: Speckle Tracking Echocardiography
