# Supplementary material for: Global longitudinal strain is a hallmark of cardiac damage in mitral regurgitation: the Italian arm of the European Registry of mitral regurgitation (EuMiClip)
Source: Cardiovasc Ultrasound. 2019 Nov 21;17:28. doi: 10.1186/s12947-019-0178-7 (PMC6873488; doi:10.1186/s12947-019-0178-7)
Supplement: Supplementary file 1 — Additional file 1: Table S1. Reproducibility of standard echocardiographic parameters [file 12947_2019_178_MOESM1_ESM.doc]

**Supplementary Table**. Reproducibility of standard echocardiographic parameters.

| Variables | ICC | 95% CI | p value |
| --- | --- | --- | --- |
| Intra-observer variability | | | |
| LV end-diastolic volume | 0.995 | 0.989- 0.998 | <0.0001 |
| LV end-systolic volume (ml) | 0.981 | 0.954-0.992 | <0.0001 |
| LVEF (%) | 0.798 | 0.495-0.919 | <0.0001 |
| LAVi (ml/m^2^) | 0.996 | 0.989- 0.998 | <0.0001 |
| PASP (mmHg) | 0.836 | 0.602-0.933 | <0.0001 |
| GLS (%) | 0.989 | 0.970-0.996 | <0.0001 |
| Inter observer variability | | | |
| LV end-diastolic volume (ml) | 0.993 | 0.981-0.997 | <0.0001 |
| LV end-systolic volume (ml) | 0.972 | 0.922-0.989 | <0.0001 |
| LVEF (%) | 0.781 | 0.470- 0.910 | <0.001 |
| LAVi (ml/m^2^) | 0.991 | 0.978- 0.996 | <0.0001 |
| PASP (mmHg) | 0.858 | 0.657-0-942 | <0.0001 |
| GLS (%) | 0.961 | 0.896-0.986 | <0.0001 |

CI = Confidence interval

ICC = Intraclass correlation coefficient

Other abbreviations as in Table 3
